# Imaging the Rovibrational Ground State of the Helium–Neon Dimers ^4^He^20^Ne and ^4^He^22^Ne

**DOI:** 10.1021/acs.jpclett.5c00377

**Published:** 2025-03-21

**Authors:** J. Kruse, J. Schröder, D. Blume, R. Dörner, M. Kunitski

**Affiliations:** † Institut für Kernphysik, Goethe-Universität, 60483 Frankfurt, Germany; ‡ Homer L. Dodge Department of Physics and Astronomy, The University of Oklahoma, Norman, Oklahoma 73019, United States; § Helmholtz Research Academy Hesse for FAIR, 64289 Darmstadt, Germany; ∥ Earth System Modelling, GFZ Helmholtz Centre for Geoscience, 14473 Potsdam, Germany

## Abstract

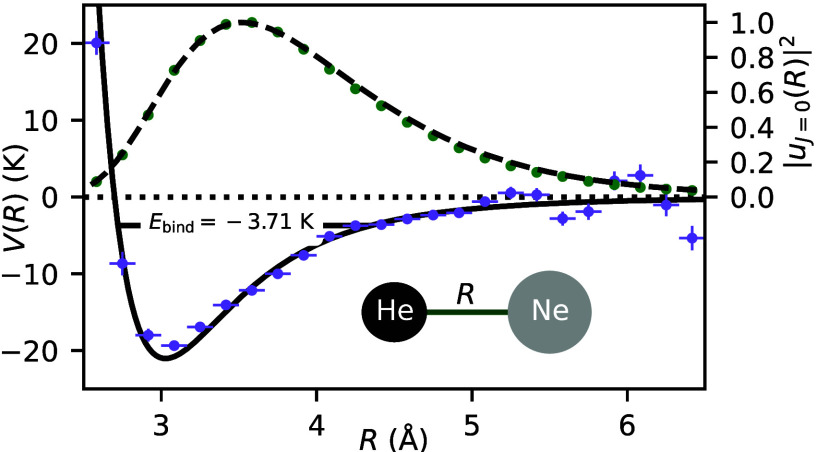

The helium–neon dimer has been subject to many theoretical studies, in which the interaction potential of the helium–neon system has been calculated with ever increasing accuracy. Calculations predict that the helium–neon system supports only a few bound states, which makes the system inaccessible to standard spectroscopic techniques. Previous experiments have probed the helium–neon potential by comparing measured and predicted scattering cross sections. However, the spatial structure and energetics of the bound states of the helium–neon system have not been studied experimentally in great detail. We employ Coulomb explosion imaging (CEI) to measure the pair distance distributions of the helium–neon dimers ^4^He^20^Ne and ^4^He^22^Ne in their rovibrational ground state. For each dimer, the binding energy is extracted from the measured pair distance distribution. Additionally, the pair distance distribution provides access to the helium–neon potential.

Small van der Waals clusters of noble gas atoms are fascinating quantum objects. The simplest system is the helium dimer ^4^He_2_, a cluster consisting of two helium atoms. Due to the small reduced mass of the system and the shallowness of the helium–helium potential, the helium dimer exists only in one single quantum state with a tiny binding energy of −1.76 mK.^1^ Consequently, the helium dimer is a huge and highly diffuse system, having a mean pair distance of 47 Å.^1^ Because 80% of the pair distance distribution resides in the classically forbidden tunnel region, the helium dimer has the characteristics of a quantum halo.^1^ Dimers consisting of heavier noble gas atoms, like neon, argon, etc., feature significantly deeper van der Waals potentials compared to the helium–helium system. This, along with their larger reduced mass, leads to the presence of many rovibrational bound states in these systems. The wave functions of the heavy dimers (and the corresponding pair distance distributions) are largely confined within the classically allowed region of the potential well. The helium–neon dimer, on the other hand, supports only very few rovibrational bound states. Therefore, it can be considered as an intermediate system bridging the realm of the helium dimer, which supports only one bound state, and the realm of the heavier noble gas clusters, which support many bound states. Dimers of weakly interacting noble gas atoms have been investigated extensively in theoretical studies. The Born–Oppenheimer potentials of homo- and heteronuclear dimers consisting of light (helium) and heavy (neon, argon, krypton, xenon, and radon) noble gas atoms have been calculated with increasing accuracy.^2−8^ Beyond Born–Oppenheimer effects have also been studied. For example, *ab initio* computations of the helium–helium potential include corrections from relativistic retardation and quantum electrodynamics.^9^ Experimentally, the interaction potentials of the various noble gas species have been probed in several ways, for example, by measuring the differential scattering cross section in crossed beam experiments^10−12^ or measuring thermophysical quantities, such as diffusion or virial coefficients.^12−14^ In this way, interaction potentials are probed rather indirectly by comparing experimental values of observables to the corresponding theoretical values derived from a theoretical potential. Other experiments have investigated the rovibrational spectra of dimers containing at least one heavy noble gas atom (neon, argon, krypton, and xenon) by various means of spectroscopy,^15−18^ some of which are isotope-sensitive.^19^ The rich rotational spectra of the heavy noble gas clusters also provide information about their geometry, because, within the rigid-rotor approximation of a diatomic molecule, the rotational constant^20^ is connected to the bond length.^16^ Although the helium–neon dimer was subject to many theoretical studies, in which the helium–neon potential^2,3,6,21^ and the corresponding rovibrational energy levels^6^ were computed, only a few experimental studies of the helium–neon dimer exist in today’s literature. Within these experiments, the helium–neon potential was probed by scattering experiments and precision measurements of virial and diffusion coefficients.^11−13^ However, to the best of our knowledge, there exist no spectroscopic studies of the helium–neon dimer in the literature. This lack of spectroscopic studies is a consequence of the low number of rovibrational bound states of the helium–neon system: According to our calculations, the helium–neon dimer exists only in the rovibrational ground state (*J* = 0) and two rotationally excited states (*J* = 1 and 2). In this experimental work, we use Coulomb explosion imaging^22−25^ to measure the pair distance distribution of the helium–neon dimer. Because Coulomb explosion imaging is isotope-sensitive, it allows distinguishing between the helium–neon dimers ^4^He^20^Ne and ^4^He^22^Ne. By comparing the measured pair distance distribution of the dimer to those from theory, we conclude that we image the rovibrational ground state of the helium–neon dimer, while the two rotationally excited states have not been populated during the dimer formation. The exponential tail of the pair distance distribution provides access to the binding energy of the dimer.^1^ In addition, we obtain the helium–neon potential from the measured pair distance distribution by inverting the time-independent Schrödinger equation.^26^

In the following, the experimental and theoretical details are described. In our experiment, helium–neon dimers form during the supersonic expansion of a mixture of 90% helium and 10% neon gas through a nozzle with an opening diameter of 5 μm. A driving pressure of 3.5 bar was chosen, and the nozzle was precooled to a temperature of 40 K. The molecular beam created by the supersonic expansion contains mostly helium or neon atoms and only ≤1% of helium–neon dimers. We mass select the helium–neon dimers from this beam by matter-wave diffraction.^27^ The same diffraction apparatus was used in previous experiments.^26,28−30^ Briefly, the molecular beam passes a skimmer of 300 μm diameter 15 mm downstream of the expansion nozzle. Subsequently, the molecular beam is collimated by a 25 μm slit, which is placed 26 mm upstream of a diffraction grating with a period of 100 nm. The mass selection is done by moving the slit and grating perpendicular to the molecular beam. In a distance of 490 mm downstream of the diffraction grating, a femtosecond laser pulse (approximately 5 μm focus diameter, 1.5 × 10^15^ W cm^–2^ intensity, 40 fs pulse duration, 780 nm central wavelength, and 8 kHz repetition rate) ionizes helium–neon dimers as they fly through the center of a COLTRIMS reaction microscope.^31^ The ions are guided to a position- and time-sensitive detector by a 10.5 V cm^–1^ electric field. The detector consists of two microchannel plates (MCPs) in Chevron arrangement followed by an hexagonal delay-line anode.^32^ This setup allows for the coincidence detection of two or more ions. The three-dimensional (3D) momentum vectors of the ions are obtained from their time of flight and their impact positions on the detector. Because the ion time of flight depends upon the ion mass, our method allows distinguishing between the helium–neon dimers ^4^He^20^Ne and ^4^He^22^Ne. A sketch of the experimental setup is shown in Figure 1a. In order to record the diffraction patterns of single helium atoms, single neon atoms, and helium–neon dimers, the slit and grating are displaced (in parallel) perpendicular to the plane of the molecular and laser beams. Figure 1b shows the diffraction patterns of single helium and neon atoms and the helium–neon dimer.

**Figure 1:**
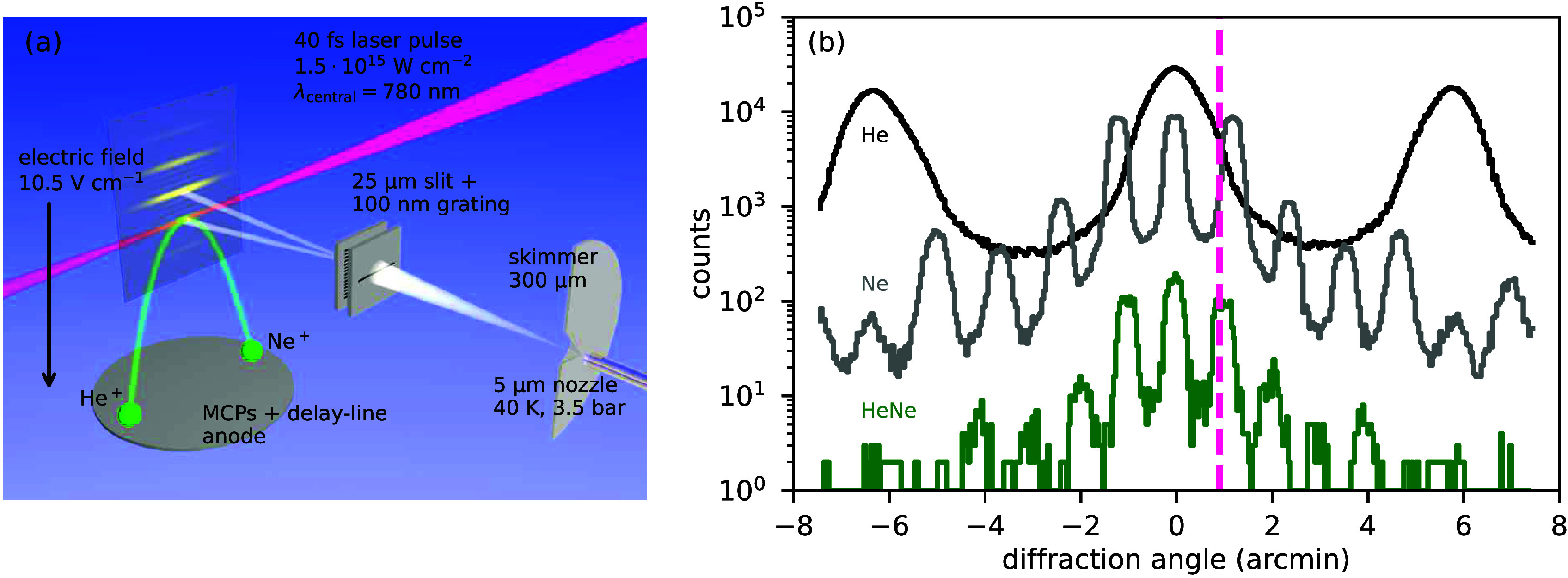
(a) Sketch of the experimental setup (see the text for details). (b) Measured diffraction patterns of single helium atoms (black curve), single neon atoms (gray curve), and helium–neon dimers (green curve). The diffraction patterns of the individual species are post-selected from the data by gating on the time-of-flight peaks of the ions He^+^, Ne^+^, and HeNe^+^, respectively. Because all species travel in the molecular beam with roughly the same mean velocity *v*_jet_, the spacing between the diffraction peaks is solely determined by the cluster mass *m* due to the de Broglie wavelength λ_dB_ = 2π/*mv*_jet_ (in the present experiment, *v*_jet_ = 600 ms^–1^ and λ_dB,^4^He_ = 0.2 nm). In order to reduce the background from the ionization of single helium and neon atoms, the laser is focused on the first-order diffraction peak of the helium–neon dimer (magenta dashed vertical line).

The pair distances of individual helium–neon dimers are measured by Coulomb explosion imaging. This technique exploits the Coulomb repulsion between the atomic ions after ionization. In the presence of the strong electric field of the laser pulse, two electrons of a helium–neon dimer are released into the continuum by tunnel ionization.^33^ As a consequence, the dimer fragments into He^+^ + Ne^+^. At the length scales relevant here, the potential energy curve of HeNe^2+^ is very well-approximated by the highly repulsive 1/*R* potential.^34^ Here, *R* is the pair distance of the dimer at the instance of ionization. During the fragmentation, the Coulomb potential energy 1/*R* of the He^+^ + Ne^+^ system is converted to the sum kinetic energy or kinetic energy release (KER) of the two fragments: KER = 1/*R* (all formulas in this work are written in atomic units). By means of this relation, the pair distance of each detected helium–neon dimer is obtained from the KER measured via the COLTRIMS reaction microscope. The pair distance distribution of ^4^He^20^Ne (^4^He^22^Ne) is acquired from 400 000 (40 000) individual measurements.

For a precise determination of the KER, a careful *in situ* calibration of the guiding electric field and the acceleration length of the ions is essential. For the calibration, we ionize O_2_ and measure the KER distribution of the fragmentation O_2_^2+^ → O^+^ + O^+^. The corresponding distribution measured by Lundquist et al.^35^ is the most accurate calibration standard in the literature. The O^+^ + O^+^ KER distribution shows a very pronounced narrow peak at 11.198 eV.^35^ We calibrate the guiding electric field and acceleration length by fine-tuning their approximate values such that the main peak of our measured O^+^ + O^+^ KER distribution is positioned at the aforementioned peak. We estimate the relative precision of this calibration to be ΔKER/KER = 6.0 × 10^–3^ = Δ*R*/*R* (where *R* is the pair distance).

From the measured pair distance distribution, the binding energy of the helium–neon dimer can be extracted. For this, we exploit the fact that the radial wave function *u*_*J*=0_(*R*) of the helium–neon dimer ground state tunnels deeply into the classically forbidden region of the potential well. For large enough pair distances, *R*, the interaction potential is almost constant and zero. Under a constant potential barrier (which is zero here), the tunneling part of the wave function *u*_*J*=0_(*R*) follows a simple exponential law^36^
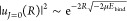
1such that the pair distance distribution depends only upon the reduced mass μ and binding energy *E*_bind_ of the dimer. Throughout this work, *E*_bind_ is considered to be negative. The binding energy of the helium–neon dimer is obtained by fitting eq 1 to the exponential tail of the measured pair distance distribution.^1,28,29^

Additionally, the pair distance distribution |*u*_*J*=0_(*R*)|^2^ of the helium–neon dimer ground state provides access to the helium–neon potential. For this, we exploit the fact that, without loss of generality, the ground state wave function of any bound system is real-valued and non-zero. Consequently, the time-independent scaled radial Schrödinger equation^36^ can be solved for the central potential.
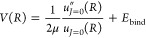
2This requires knowledge of the scaled radial wave function *u*_*J*=0_(*R*), which satisfies the normalization ∫_0_^*∞*^|*u*_*J*=0_(*R*)|^2^ d*R* = constant, and its second derivative *u*_*J* = 0_^″^(*R*). The ground-state wave function *u*_*J*=0_(*R*) is connected to the pair distance distribution via *N*(*R*) ≡|*u*_*J*=0_(*R*)|^2^ (number of counts at pair distance *R*), such that eq 2 can be rewritten as
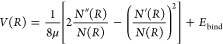
3where the first and second derivative *N*′(*R*) and *N*″(*R*) are obtained by numerical differentiation of the measured pair distance distribution. This method of potential determination assumes only the validity of the time-independent Schrödinger equation and does not rely on any assumption about the theoretical form of the potential.^26^

The results are discussed in the following section. In order to compare our measurements to theory, we calculate the bound-state wave functions and binding energies of the helium–neon dimers ^4^He^20^Ne and ^4^He^22^Ne from the theoretical helium–neon potential of Cacheiro et al.^6^ To determine the eigenstates and eigenenergies of the time-independent Schrödinger equation for the aforementioned potential, we solve the scaled radial Schrödinger equation for the reduced mass μ separately for each *J*. The angular kinetic energy introduces the angular momentum barrier^36^
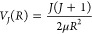
4into the radial equation. The radial equation is solved by expanding the eigenstates in terms of a B-spline basis using a nonlinear grid in *R* (more points in the small-*R* region and fewer points in the large-*R* region). Between 1000 and 4000 grid points are used. The values of *R*_min_ and *R*_max_, at which the scaled radial wave function *u*_*J*_(*R*) is forced to vanish, are varied to ensure convergence of the bound eigenstates and eigenenergies with regard to the numerical box size. Using this approach, we find three rovibrational bound states for each of the two dimers ^4^He^20^Ne and ^4^He^22^Ne. These are the three rotational states *J* = 0, 1, and 2 of the vibrational ground state ν = 0. In their study, Cacheiro et al. predicted an additional vibrationally excited bound state with *J* = 0 and ν = 1, which is located in the vicinity of the dissociation threshold. Conducting thorough cross-checks of our calculation, we could not confirm the existence of this fourth bound state. Table 1 compares the experimental and theoretical binding energies of the dimers ^4^He^20^Ne and ^4^He^22^Ne.

**Table 1:** Binding Energies *E*_ν,*J*_ of ^4^He^20^Ne and ^4^He^22^Ne in Kelvin^a^

*E*_ν,*J*_		this work (experiment)	this work^6^ (theory)	Cacheiro^6^ (theory)	Cybulski^8^ (theory)	Ogilvie^37^ (theory)
*E*_0,0_	^4^He^20^Ne	–3.71 ± 0.15	–3.7138^b^	–3.7138^c^	–3.6981^b^ (−3.6978^c^)	–3.6075^b^
	^4^He^22^Ne	–4.6 ± 0.8	–3.7878^b^			
*E*_0,1_	^4^He^20^Ne		–2.6927^b^	–2.6930^c^	–2.6778^b^	–2.5916^b^
	^4^He^22^Ne		–2.7762^b^			
*E*_0,2_	^4^He^20^Ne		–0.7647^b^	–0.7653^c^	–0.7519^b^	–0.6732^b^
	^4^He^22^Ne		–0.8605^b^			
*E*_1,0_	^4^He^20^Ne		_	–0.0001^c^	_	_
	^4^He^22^Ne		_			

a ν is the vibrational quantum number, and *J* is the quantum number of total angular momentum. Theoretical binding energies of this work are computed from the potential of Cacheiro et al.^6^ Blank cells indicate that the binding energy has not been measured or calculated, while dashes indicate that the bound state was not found in the calculation.

b Calculated by using the potential from the paper.

c Directly taken from the paper.

For the dimer ^4^He^20^Ne, the calculated pair distance distributions |*u*_*J*_(*R*)|^2^ of the three bound states *J* = 0, 1, and 2 are shown in Figure 2a by lines along the measured one (symbols with error bars). The corresponding effective potentials of the three states are depicted in Figure 2b. They differ by the repulsive centrifugal barrier (eq 4). As a consequence, at larger pair distances, the pair distance distributions |*u*_*J*_(*R*)|^2^ of the rotationally excited states differ significantly from that of the rovibrational ground state (see Figure 2a). The measured pair distance distribution is in good agreement with the theoretical pair distance distribution |*u*_*J*=0_(*R*)|^2^ of the rovibrational ground state. Thus, we conclude that, during dimer formation, the helium–neon dimer is prepared in the rovibrational ground state, while the population of the higher *J* states is negligible. This implies, given the theoretical binding energies, that the effective rotational temperature is below 0.5 K.

**Figure 2:**
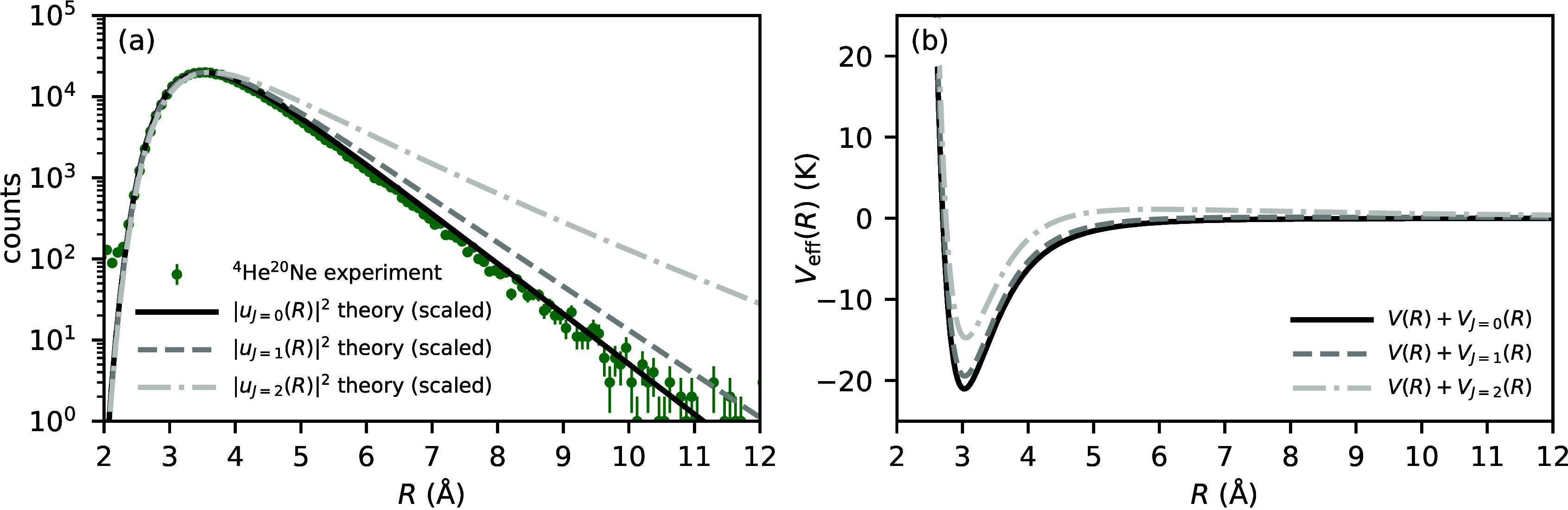
(a) Green dots with error bars: measured pair distance distribution of ^4^He^20^Ne. Error bars correspond to the statistical error √*N*, where *N* is the number of counts per bin. Solid, dashed, and dash-dotted lines: computed pair distance distributions |*u*_*J*_(*R*)|^2^ of the three helium–neon dimer bound states *J* = 0, 1, and 2 based on the interaction potentials (b). The distributions |*u*_*J*_(*R*)|^2^ are scaled to the experimental distribution. (b) Sum of the helium–neon potential^6^ and repulsive centrifugal barrier (eq 4).

Figure 3 shows the measured pair distance distributions of the helium–neon dimers ^4^He^20^Ne and ^4^He^22^Ne. The dimer ^4^He^20^Ne is detected roughly 10 times more often than the isotope-substituted dimer ^4^He^22^Ne due to the difference in the natural abundance of the isotopes ^20^Ne and ^22^Ne. At pair distances *R* ≤ 5 Å, within the strongly attractive part of the interaction potential, the shape of the pair distance distributions of ^4^He^20^Ne and ^4^He^22^Ne is almost identical. The exponentially decaying parts of the distribution (*R* > 5.5 Å) are slightly different however. This relates to the binding energy of the dimer ground state and the reduced mass, as pointed out above (eq 1). The rovibrational ground states of ^4^He^20^Ne and ^4^He^22^Ne, which we computed from the helium–neon potential of Cacheiro et al.,^6^ have binding energies of −3.71 and −3.79 K, respectively. Correspondingly, for both dimer species, the classical turning point *R*_turn_ of the potential lies at approximately at 4.2 Å. Beyond the classical turning point, the radial wave function of the helium–neon dimer tunnels into the classically forbidden potential well. At pair distances larger than 5.5 Å, the potential is almost constant and zero, such that the tunneling wave function follows the exponential form in eq 1. By fitting eq 1 to the pair distance distributions in Figure 3, we obtain binding energies of (−3.71 ± 0.15) K for ^4^He^20^Ne and (−4.6 ± 0.8) K for ^4^He^22^Ne. The values of the fitting parameters are slightly dependent upon the *R* region in which the fit is done. We obtained the binding energies and corresponding errors by performing 30 fits, where the lower boundary of the fit was systematically modified in the range of 5.5–8.0 Å while fixing the upper boundary at 10.0 Å for ^4^He^20^Ne and 9.0 Å for ^4^He^22^Ne, respectively. The specified values of the binding energy and uncertainty correspond to median binding energy and median uncertainty obtained from all 30 fits. While, for ^4^He^20^Ne, the measured binding energy (−3.71 ± 0.15) K is consistent with the theoretical value of −3.71 K, for the isotope-substituted dimer ^4^He^22^Ne, the theory value of −3.79 K is 1.1σ away from the measured value of (−4.6 ± 0.8) K. Within the Born–Oppenheimer approximation, the difference in the binding energies between isotope-substituted species is only determined by the difference in their reduced mass. In the current case, the binding energies differ by about 2% (−3.71 K for ^4^He^20^Ne versus −3.79 K for ^4^He^22^Ne). Adiabatic and non-adiabatic corrections to the Born–Oppenheimer potentials have been quantified extensively for small systems (e.g., HeH^+^)^38^ but much less so for heavier systems (e.g., Ne_2_ or H_2_O).^39,40^ Based on these results, we estimate that changes of the binding energies that arise from corrections to the Born–Oppenheimer potential are smaller than the experimental error bars reported in this work. Thus, the mismatch of 1.1σ between the experimental and theoretical binding energy of ^4^He^22^Ne cannot be explained using a more complete potential beyond the Born–Oppenheimer approximation.

**Figure 3:**
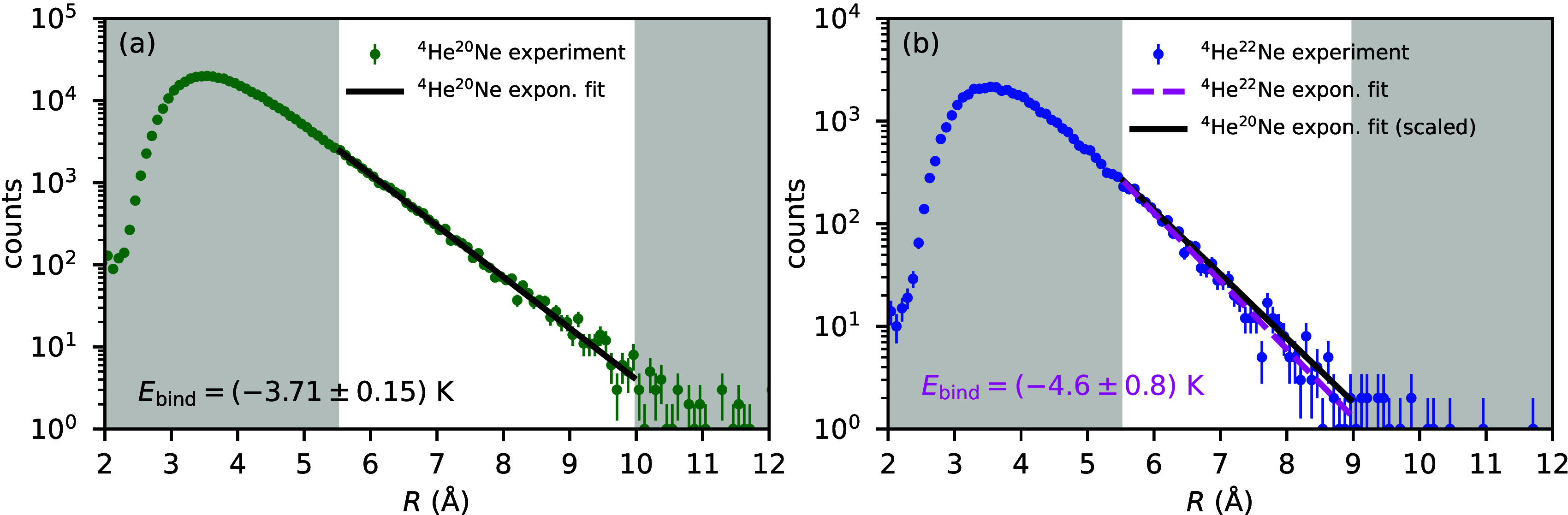
(a) Measured pair distance distribution of ^4^He^20^Ne. (b) Measured pair distance distribution of ^4^He^22^Ne. Error bars correspond to the statistical error √*N*, where *N* is the number of counts per bin. Note the different ranges of the *y* axis in panels a and b. The binding energy of each dimer is obtained from a fit of the exponential tail of the pair distance distribution. The binding energies that we computed from the potential of Cacheiro et al.^6^ are −3.71 K for ^4^He^20^Ne and −3.79 K for ^4^He^22^Ne.

Because the measured pair distance of ^4^He^20^Ne corresponds to one single bound state and not a superposition or statistical mixture of two or more states, it can be used to extract the helium–neon potential via the time-independent Schrödinger equation (eq 3). Figure 4 shows the helium–neon potential derived from the measured pair distance distribution of ^4^He^20^Ne. Overall, the experimental data points resemble the theoretical potential of Cacheiro et al.^6^ quite well. While measurements of differential cross sections in scattering experiments probe the weakly repulsive well of the helium–neon potential, measurements of virial coefficients probe the potential minimum and the attractive tail of the potential.^12^ Our method, in contrast, allows us to probe the potential landscape on the entire region where the pair distance distribution is non-zero. Furthermore, experimental methods based on differential scattering cross sections and thermophysical quantities typically rely on the choice of the theoretical interaction potential. Therefore, these methods are never assumption-free. Our method, on the other hand, relies on the clean preparation of the dimer ground state, but from the theory point of view, it only assumes the validity of the time-independent Schrödinger equation, while no guess about the particular shape of the interaction potential is required. This method has previously been successfully applied for mapping the interaction potentials of He_2_, Ne_2_, Ar_2_, and H_2_.^26^

**Figure 4:**
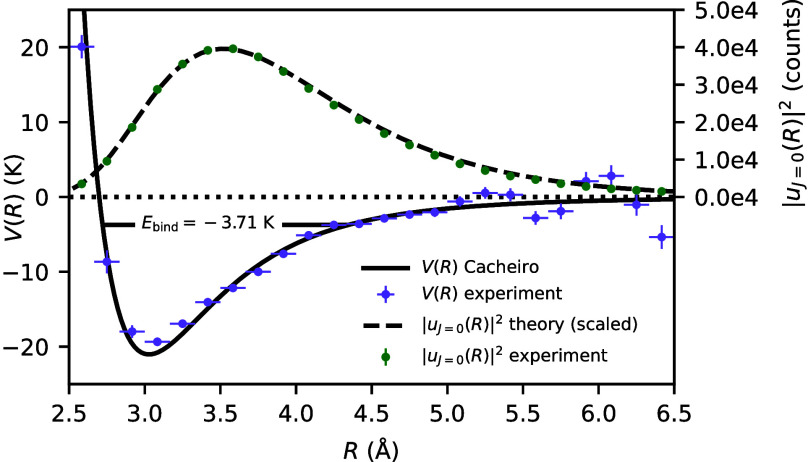
Purple dots represent the helium–neon potential computed from the measured pair distance distribution of ^4^He^20^Ne via eq 3. Horizontal error bars correspond to the bin width Δ*R*. For each data point of the measured potential curve, the vertical error bar is derived from eq 3 by computing a Gaussian error propagation for the statistical error √*N* of the pair distance distribution (green dots). The black solid line corresponds to the theoretical helium–neon potential of Cacheiro et al.^6^

In summary, we used Coulomb explosion imaging to measure the pair distance distributions of the helium–neon dimers ^4^He^20^Ne and ^4^He^22^Ne in their rovibrational ground state. The binding energies of the two species were obtained from the exponential tail of the measured pair distance distributions. The experimental observations are in good agreement with theory calculations based on the theoretical helium–neon potential of Cacheiro et al.^6^ However, using this potential in our calculations, we cannot confirm the existence of the vibrationally exited bound state, although the theoretical binding energies of the states *J* = 0, 1, and 2 are in good agreement with those reported by Cacheiro et al.^6^ In addition, the helium–neon potential was extracted from the measured pair distance distribution. It agrees with the aforementioned theoretical potential^6^ within the error bars. As a system supporting only three bound states, the helium–neon dimer can be considered as an intermediate system between the single bound state of the helium dimer, which is ultraweakly bound, and the more strongly bound noble gas clusters containing the heavier noble gas atoms (neon, argon, krypton, xenon, and radon). While this work is focused on the stationary properties of the helium–neon dimer, it would be interesting to observe how this system, which supports only a few rotational bound states, responds to a non-adiabatic laser kick. The laser-induced dynamics might show characteristics of the rotational dynamics of the helium dimer^30^ as well as the dynamics of the heavier noble gas dimers, which typically show rotational revivals.^41−43^

## Supporting Information Available

Transparent Peer Review report available (PDF)
