# Supplementary material for: Imaging the Rovibrational Ground State of the Helium–Neon Dimers 4He20Ne and 4He22Ne
Source: J Phys Chem Lett. 2025 Mar 21;16(13):3225–31. doi: 10.1021/acs.jpclett.5c00377 (PMC11973920; doi:10.1021/acs.jpclett.5c00377)
Supplement: Supplementary file 1 — jz5c00377_si_001.pdf [file jz5c00377_si_001.pdf]

jz-2025-003774.R1

Name: Peer Review Information for "Imaging the rovibrational ground state of the helium-neon dimers 4He20Ne and 4He22Ne"

First Round of Reviewer Comments

Reviewer: 1

Comments to the Author

Review of:” Imaging the rovibrational ground state of the helium-neon dimers $^{4}He^{20}Ne$ and $^{4}He^{22}Ne$”. Manuscript ID: jz-2025-003774

This manuscript presents innovative results about the usage of the Coulomb explosion imaging technique for the characterization of the $^{4}He^{20}Ne$ and $^{4}He^{22}Ne$ dimers in terms of binding energies and pair distance distribution, leading to a deduction of the $^{4}He^{20}Ne$ ground state potential energy curve. Comparisons between theory and experiment show great agreement and accordance with published data. The manuscript is well-written, clear, and straightforward. The results are of high importance to the readership, given their novelty and accuracy. I recommend the publication of the manuscript with very minor comments proposed below **Minor Revisions:**

1) Highlighting sections within the document would provide more clarity to the readership.
2) Adding references explaining equations (1), (2), and (4) would ease the understanding of the text.
3) Page 7, line 50: A few lines explaining the method to solve the time-independent Schrodinger equation would provide a better assimilation of the proposed ideas.
4) Line 48 p11-“Summary” not “summery”

Reviewer: 2

Comments to the Author

The authors introduced a novel approach for determining the binding energy of weakly bound heteroatomic complexes, such as of helium and neon atoms. The complexes are produced via supersonic jet expansion of the He/Ne mixture and isolated via the matter wave diffraction. The complexes are irradiated by strong femtosecond pulses yielding He^+ and Ne^+ ions that experience Coulomb repulsion. The kinetic energy released is obtained using a coincidence detection in so called COLTRIMS reaction microscope experiment. This allows to trace back the distance between the atoms, R, before the laser irradiation and upon observation of many events reconstruct the radial wavefunction of the dimers and the interaction potential. The asymptotic part of the wavefunction at large R decays exponentially which enables to attain an accurate binding energy of the dimers. This work reports the first experimental values for the binding energy of the He-20Ne dimers which is good agreement with previous calculations. The manuscript is well written and should be published in JPCL upon revision as specified in the following.

1. The dimers are expected to have three bound states. The authors concluded that only the ground state is populated. This is a critical assumption, because if several states are populated the obtained binding energy will be a weighted average of the three states. The authors should discuss the effect of the unknown fraction of the excited states on the accuracy of the determined binding energy.

2. The first excited rotational state has energy of about 1 K. If the temperature of the jet is 1 K the population of the excited state would be about the same as of the ground state. Much lower jet temperature is required for the ground state to have the dominant population. I wonder if such low temperature is reasonable. For example, the formation of the clusters in the jet leads to higher temperatures as compared the estimates based on the adiabatic expansion. The authors should present a realistic estimate of the temperature of the complexes to support their assumptions.

3. Whereas the binding energy of the He-20Ne complexes is in good agreement with the results of the calculations, for the He-22Ne they report about 1 K larger binding which is in disconcert with the calculations, that show just 0.07 K difference. The authors stated that the possible adiabatic and non-adiabatic corrections to the Born-Oppenheimer potentials could not explain the difference. I found that the large difference between the binding of the two isotopes, which is usually well tractable, is concerning. I suggest the authors outline possible experimental reasons for this difference.

Author's Response to Peer Review Comments:

Dear Editors of The Journal of Physical Chemistry Letters,

We resubmit the revised version of our manuscript "Imaging the rovibrational ground state of the heliumneon dimers 4He20Ne and 4He22Ne" (ID jz-2025-003774) after it was assessed by two referees. We would like to thank the referees for their thorough reading and valuable comments and suggestions. In the following we address all points of criticism and answer the questions raised.

We hope that the revised manuscript will now be accepted for publication in the Journal of Physical Chemistry Letters.

Best regards,

The authors

(J. Kruse, J. Schröder, D. Blume, R. Dörner, M. Kunitski)

Response to the referee comments and summary of the changes to the manuscript

---

In the following, the comments of the referees are cited in italic blue font, while our answers to these comments are written in black upright font.

Reviewer: 1

Review of:" Imaging the rovibrational ground state of the helium-neon dimers 4He20Ne and 4He22Ne". Manuscript ID: jz-2025-003774

This manuscript presents innovative results about the usage of the Coulomb explosion imaging technique for the characterization of the 4He20Ne and 4He22Ne dimers in terms of binding energies and pair distance distribution, leading to a deduction of the 4He20Ne ground state potential energy curve. Comparisons between theory and experiment show great agreement and accordance with published data. The manuscript is well-written, clear, and straightforward. The results are of high importance to the readership, given their novelty and accuracy. I recommend the publication of the manuscript with very minor comments proposed below Minor Revisions:

We thank the referee for the precise summary and positive assessment of our work. Furthermore, we thank the referee for the constructive suggestions, which are all addressed in the revised version of the manuscript:

1. *Highlighting sections within the document would provide more clarity to the readership*

   While the journal does not allow section headlines, we added introduction sentences in order to mark the beginnings of the sections on the experimental methods and the final results. The introduction sentences should increase the readability of the text.

2. *Adding references explaining equations (1), (2), and (4) would ease the understanding of the text*

   To all three equation mentioned above, we added a reference to the Quantum mechanics textbook of Griffiths, in order to provide the reader with some context about these equations.

3. *3Page 7, line 50: A few lines explaining the method to solve the time-independent Schrodinger equation would provide a better assimilation of the proposed ideas.*

   On page 8 we added a short description of the numerical methods which we used in order to solve the time-independent Schrödinger equation.

4. *Line 48 p11-"Summary" not "summery"*

   We thank the referee for catching this typo!

Reviewer :2

The authors introduced a novel approach for determining the binding energy of weakly bound heteroatomic complexes, such as of helium and neon atoms. The complexes are produced via supersonic jet expansion of the He/Ne mixture and isolated via the matter wave diffraction. The complexes are irradiated by strong femtosecond pulses yielding He+ and Ne+ ions that experience Coulomb repulsion. The kinetic energy released is obtained using a coincidence detection in so called COLTRIMS reaction microscope experiment. This allows to trace back the distance between the atoms, R, before the laser irradiation and upon observation of many events reconstruct the radial wavefunction of the dimers and the interaction potential. The asymptotic part of the wavefunction at large R decays exponentially which enables to attain an accurate binding energy of the dimers. This work reports the first experimental values for the binding energy of the He-20Ne dimers which is good agreement with previous calculations. The manuscript is well written and should be published in JPCL upon revision as specified in the following.

We thank the referee for the precise and detailed summary of work. Furthermore, we thank the referee for pointing out the relevance of our manuscript.

1. *The dimers are expected to have three bound states. The authors concluded that only the ground state is populated. This is a critical assumption, because if several states are populated the obtained binding energy will be a weighted average of the three states. The authors should discuss the effect of the unknown fraction of the excited states on the accuracy of the determined binding energy.*

   The referee is right that the relative population of these three bound states is highly important for determination of binding energies and even more crucial for retrieval of the potential from the measured pair distance distribution. Our conclusion that only the ground state is populated based on the comparison of the measured pair distance distribution with theoretical ones (Figure 2a). The exponential tails of these distributions (R>6Å) look different (see Figure 2a), since they are determined by the corresponding binding energy. The measured distribution resembles very close that of the ground state. The following sentences summarize this in the manuscript: “The measured pair distance distribution is in good agreement with the theoretical pair distance distribution |uJ=0(R)|2 of the rovibrational ground state. Thus, we conclude that during dimer formation, the helium-neon dimer is prepared in the rovibrational ground state, while the population of the higherJ states is negligible.”

   Moreover we have tried to fit the measured distribution with an incoherent sum of three theoretical distributions optimizing the corresponding weights, i.e. populations, as it would be a statistical mixture of all three states. However the best

fit have resulted in zero populations for excited J=1 and J=2 states (see the green line below). For instance, the resulted pair distance distribution for the case, where J=1 and J=2 states were populated by 10% and 5%, respectively, is depicted below in blue.

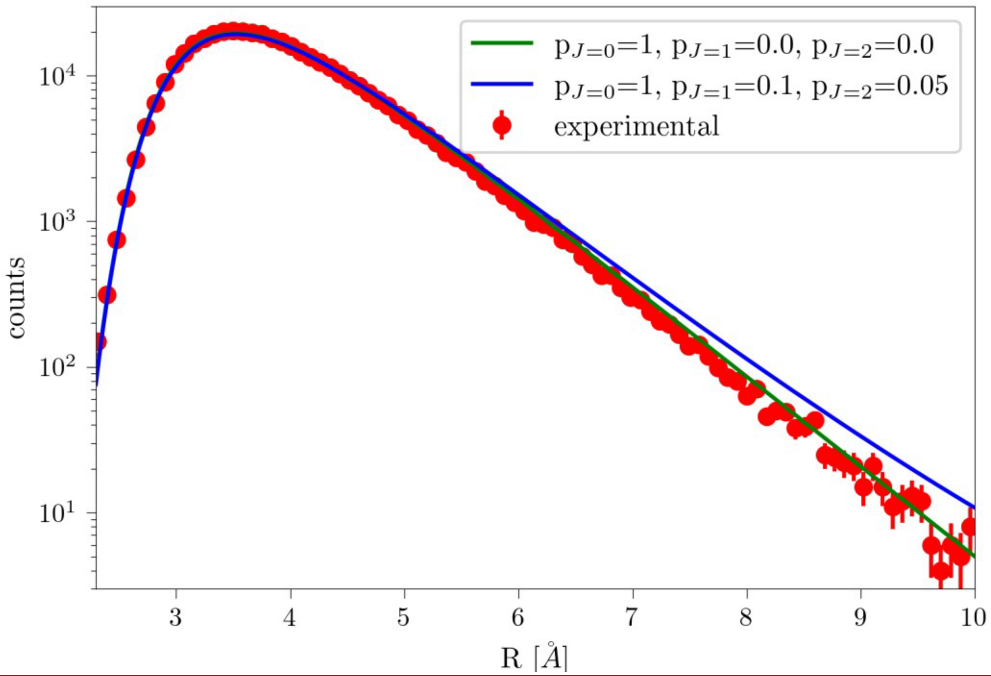

Figure R1. The experimental pair distance distribution of $^4He^{20}Ne$ and fits using theoretical distributions and different state populations.

There is also an argument, which doesn't require knowledge of theoretical pair distance distributions. Namely, the population of more than one state would result in a non-exponential decay of the measured pair distance distribution (as, for instance, seen in Fig. R1 in the case of blue distribution).

2. *The first excited rotational state has energy of about 1 K. If the temperature of the jet is 1 K the population of the excited state would be about the same as of the ground state. Much lower jet temperature is required for the ground state to have the dominant population. I wonder if such low temperature is reasonable. For example, the formation of the clusters in the jet leads to higher temperatures as compared the estimates based on the adiabatic expansion. The authors should present a realistic estimate of the temperature of the complexes to support their assumptions.*

This is a very interesting question, and it certainly relates to the previous one. During expansion commonly three different temperatures: vibrational, rotational and translational are used in order to describe population of corresponding levels. These three temperatures are usually quite different, since the cooling rate during expansion depends on the spacing between levels. The larger the spacing is, the less efficient states get depopulated during collisions. Under typical expansion conditions starting at 300 K one can cool down the rotational temperature below 10 K [Appl. Phys. B 52, 84–89 (1991)] and under extreme conditions using high stagnation pressures of a carrier gas even below 1 K [J. Chem. Phys. 112, 8068–8071 (2000), J. Chem. Phys. 118, 8699–8705 (2003)]. Our case is however different. There are no rotational degrees of freedom prior to expansion, since dimers are formed during expansion. It was shown, for instance, that formation of Ar_2 with a stagnation temperature of 300 K results in a rotational temperature of few Kelvins (2 - 4.7 K, Phys. Rev. A 83, 061403(R)). In our case the stagnation temperature is 40 K, which implies that even lower rotational temperatures can be reached (giving the same cooling rate).

Another argument, which allows to justify the low *rotational* temperature is the extremely low *translational* temperature, which is achieved during helium expansion (our gas mixture consists to 90% of helium). In J. Chem. Phys. 117, 1544–1566 (2002) for similar expansion conditions (30K, 2-20bar) of helium *translational* temperatures below 5mK are reported.

In addition, we still think that our experimental pair distance distribution can serve as a
"thermometer" for estimation of the rotational temperature. For a given rotational temperature we calculate populations for all three state and construct an overall distribution using theoretical ones in a similar way as shown above in Fig. R1.

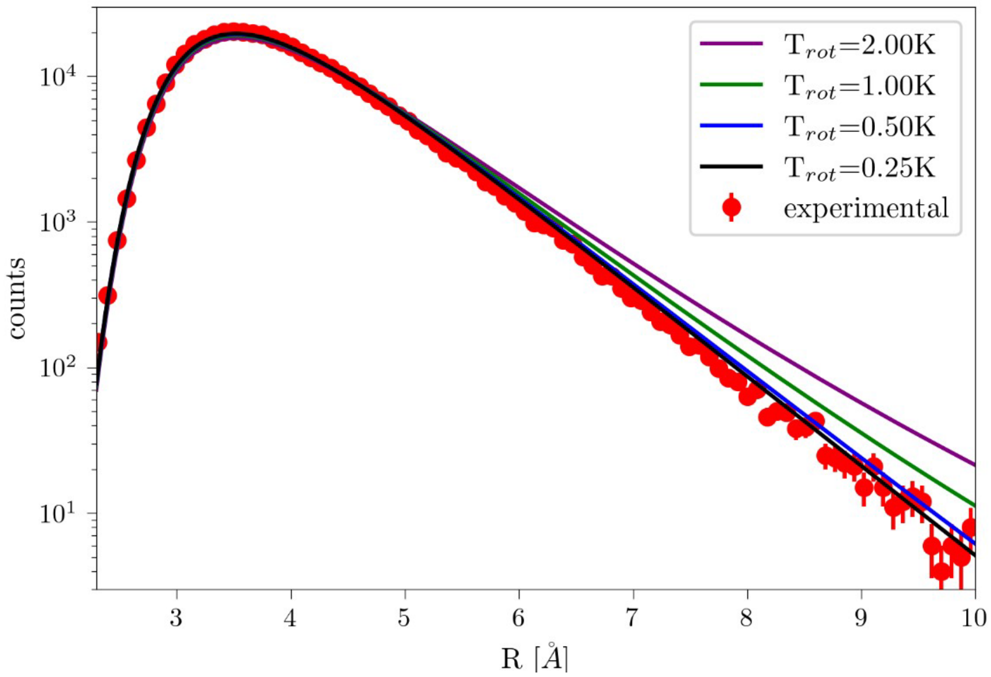

Figure R2. Dependence of the pair distance distribution of $^4He^{20}Ne$ on the effective rotational temperature. The distribution for T_{rot}=0K (not shown) cannot be distinguished from one with T_{rot}=0.25K on the scale of the plot.

Comparing these simulations to the experimental distribution one can conclude that the effective rotational temperature is below 0.5 K.

In order to address this concern we have added the following sentence to the manuscript: “This implies, given the theoretical binding energies, that the effective rotational temperature is below 0.5 K.”

3. Whereas the binding energy of the He-20Ne complexes is in good agreement with the results of the calculations, for the He-22Ne they report about 1 K larger binding which is in disconcert with the calculations, that show just 0.07 K difference. The authors stated that the possible adiabatic and non-adiabatic corrections to the Born-Oppenheimer potentials could not explain the difference. I found that the large difference between the binding of the two isotopes, which is usually well tractable, is concerning. I suggest the authors outline possible experimental reasons for this difference.

The referee is correct that the difference of the theoretical binding energies is only 0.07 K for 4He20Ne and 4He22Ne. This difference, however, is still within the error bars of the measured values:

$$(4.6 - 0.8)\ \text{K} – (3.71 + 0.15)\ \text{K} = 0.06\ \text{K}.$$

As pointed out in the manuscript, for 4He22Ne the theoretical binding energy is 1.1 sigma away from the measured value. This suggests that the deviation between theory and experiment is not statistically significant and, within error bars, there is still agreement of experiment and theory. Thus, we argue that for 4He22Ne the 1.1 sigma deviation between experiment and theory is not related to physical effects but rather to the statistics of the 4He22Ne data, which is ten times lower than the statistics of 4He20Ne.
